# PSMC2/CCND1 axis promotes development of ovarian cancer through regulating cell growth, apoptosis and migration

**DOI:** 10.1038/s41419-021-03981-5

**Published:** 2021-07-22

**Authors:** Dawei Zhu, Jie Huang, Ning Liu, Wei Li, Limei Yan

**Affiliations:** 1 Department of Gynaecology and Obstetrics, Daping Hospital, Army Medical University, Chongqing, 400042 China; 2 Department of Obstetrics and Gynaecology, Shengjing Hospital of China Medical University, Heping District, Shenyang, 110004 Liaoning China

**Keywords:** Gynaecological cancer, Gynaecological cancer

## Abstract

Ovarian cancer is known as one of the most common malignancies of the gynecological system, whose treatment is still not satisfactory because of the unclear understanding of molecular mechanism. PSMC2 is an essential component of 19 S regulatory granules in 26 S proteasome and its relationship with ovarian cancer is still not clear. In this study, we found that PSMC2 was upregulated in ovarian cancer tissues, associated with tumor grade and could probably predict poor prognosis. Knocking down the endogenous PSMC2 expression in ovarian cancer cells could decrease colony formation ability, cell motility and cell proliferation rate, along with increasing cell apoptosis rate. Cells models or xenografts formed by cells with relatively lower expression of PSMC2 exhibited weaker oncogenicity and slower growth rate in vivo. Moreover, gene microarray was used to analyze the alteration of gene expression profiling of ovarian cancer induced by PSMC2 knockdown and identify CCND1 as a potential downstream of PSMC2. Further study revealed the mutual regulation between PSMC2 and CCND1, and demonstrated that knockdown of CCND1 could enhance the regulatory effects induced by PSMC2 knockdown and overexpression of CCND1 reverses it. In summary, PSMC2 may promote the development of ovarian cancer through CCND1, which may predict poor prognosis of ovarian cancer patients.

## Introduction

Ovarian cancer is the gynecological malignant tumor with high mortality rates, of which epithelial ovarian cancer is the most important pathological types [1]. Ovarian cancer is one of the three considerable malignancies of the female reproductive system. It is characterized by complex histopathology, high degree of malignancy and poor survival prognosis. It has also been considered one of the main causes of cancer-related death in women. Epidemiological studies showed that in 2015, there were about 52000 new cases of ovarian cancer and 23000 deaths in China [2, 3]. Moreover, the mortality/incidence ratio of ovarian cancer in China fluctuated around 0.43, which is significantly higher than that of other gynecological tumors [3]. The onset of ovarian cancer is insidious and it progresses rapidly. Therefore, most patients are in advanced stage by the time they were initially diagnosed [4]. Unfortunately, it has been well-documented that surgical treatment is only effective for patients in early stage, and most patients with advanced disease are not sensitive to conventional chemotherapy and radiotherapy [5, 6]. Currently, molecular targeted drugs, targeting the inhibition of vascular endothelial growth factor (VEGF), epidermal growth factor receptor (EGFR), platelet derived factor receptor (PDGFR) and so on, shed light on the treatment of ovarian cancer [7, 8]. Therefore, in-depth study of the molecular mechanism of ovarian cancer development and drug resistance, from which to develop effective treatment strategies, is the problem that must be overcome in the way to precision treatment of ovarian cancer.

Ubiquitin-proteasome system (UPS) is one of the most well-known protein degradation pathways. Ubiquitin mediated protein degradation is a specific pathway of protein degradation through proteasome in cells, which is strictly regulated by time and space [9, 10]. The most common structural form of proteasome in UPS is 26 S proteasome, with a molecular weight of about 2000 kDa. It contains a core particle of 20 S in the center and two regulatory particles of 19 S on both sides or one side [11–13]. The 20 S core particles have hollow structure and possess potential catalytic activity, which need to be activated. At least two kinds of proteasome activators have been identified, which can interact with 20 S proteasome and enhance its catalytic activity [14, 15]. These two kinds of activators are 11 S regulatory granules and 19 S regulatory granules. 19 S regulatory granules are composed of different subunits and contain 19 protein components, each of which has its own unique regulatory effects on the whole proteasome structure [15, 16]. Proteasome 26 S subunit ATPase 2 (PSMC2) is one of the essential components of 19 S subunit. It has the functions of ATP binding, nucleotide binding, nucleoside triphosphatase and hydrolase. It is mainly involved in regulating the selective degradation of intracellular proteins [17]. Recently, more and more attention has been paid on the biological functions of PSMC2 in the development and progression of human cancers such as pancreatic cancer and osteosarcoma [18, 19]. However, the relationship between PSMC2 and ovarian cancer has been seldom studied and remained unknown.

This study was accomplished for the purpose of getting insight into the role and mechanism of PSMC2 in ovarian cancer development. We observed higher expression of PSMC2 in ovarian cancer tissues than normal tissues. Survival analysis based on data collected from TCGA indicated the potential linkage between PSMC2 high expression and poor prognosis of patients with ovarian cancer. In vitro and in vivo assays elucidated that silencing PSMC2 could significantly inhibit ovarian cancer development through regulating cell growth, apoptosis and migration. Moreover, preliminary mechanistic study revealed that PSMC2 may execute its regulatory effects on ovarian cancer in combination with CCND1. In a word, our work identified PSMC2 as a critical participate in ovarian cancer development, which could be a target for diagnosis or treatment of ovarian cancer. Moreover, considering that CCND1 plays essential roles in current therapeutic strategies for ovarian cancer patients that involve platinum therapy and checkpoint inhibitors, the exploration of the functions of PSMC2 and CCND1 in ovarian cancer should be of great significance.

## Materials and Methods

### Cell culture

Human ovarian cancer cell lines OVCAR-3 and HO-8910 were purchased from the Institute of Biochemistry and Cell Biology of the Chinese Academy of Sciences (Shanghai). OVCAR-3 and HO-8910 cells were cultured in 90% RPMI 1640 (Corning) supplemented with 10% FBS. Cells were cultured in a carbon dioxide incubator (SANYO) with 5% CO_2_ at 37 °C.

### Immunohistochemical (IHC) staining

Human ovarian cancer tumor tissues and adjacent normal tissues microarray (Cat. # Hovac160Su01) was purchased from Xi’an Alena Biotech Co., Ltd., which were collected from patients with ovarian cancer. The experiment was approved by ethical committee of Shengjing Hospital of China Medical University. Written informed consent and pathological data of each patient was collected. For IHC assay, microarrays were dewaxed with dimethylbenzene and dehydrated with gradient alcohol (100%, 90% and 75%). After antigen retrieval with citrate buffer, blocked with 3% H_2_O_2_, and incubated with corresponding serum, slides were incubated with anti-PSMC2 (1:100, Cat. # SC-166972, SANTA CRUZ) and anti-CCND1 (1:250, Cat. # ab40754, Abcam) at 4 °C overnight, following incubated with appropriate horseradish peroxidase (HRP)-conjugated IgG polyclonal antibody for 30 min at room temperature. DAB and hematoxylin were used for slides staining. IHC scoring of specimens were determined derived from the sum of the staining intensity and staining extent scores.

### Lentiviral vector construction

RNA interference of target gene human PSMC2 (shPSMC2) and CCND1 (shCCND1) were designed by Shanghai Yibeirui Bioscienceres, Co., Ltd. and the sequences were showed in Table S1. shRNA sequences were reverse transcript to cDNA and double stranded DNA were synthesized. Linearized double stranded DNA was transformed into competent E. coli cells and cultured overnight, positive clones were identified by PCR. The sequenced bacterial solution was cultured and plasmid was extracted using EndoFree Maxi Plasmid Kit (Cat. # DP118-2, TIANGEN). Purified plasmid with target sequences were co-transfected into 293 T cells with BR-V-108, Helper 1.0 and Helper 2.0. After cultured for 72 h, lentiviruses were collected, and lentivirus were concentrated and purified for viral packaging. Empty lentiviral vector was used as control. For overexpressing CCND1, the CCND1 construct was generated by subcloning human CCND1 cDNA into vector (Shanghai Yibeirui Bioscienceres, Co., Ltd.) and empty vector was used as the negative control.

### Cell infection

HO-8910 and OVCAR-3 cells were seeded into 6-well plates with 2×10^5^ and cultured in RPMI 1640 medium at 37 °C. 400 μL infective fluid including ENI.S plus Polybrene and 1 × 10^7^ TU/well lentivirus was added into each well for cell infection. After 72 h culturing, cells were observed using fluorescence microscope (OLYMPUS) and the fluorescence efficiency were estimated.

### RT-qPCR

Total RNAs in lentivirus infected HO-8910 and OVCAR-3 cells were extracted with Trizol Reagent (Sigma). Then the concentration of total RNA was determined by Nanodrop 2000/2000c spectrophotometer (Thermo). cDNA was obtained by RNA reverse transcription using Hiscript QRT supermix for qPCR (+gDNA WIPER) (Vazyme). PCR reaction system was prepared with SYBR Premix Ex Taq and suitable primers (the primer sequences were detailed in Table S2). Real time PCR was performed by two steps, melting curve was made, and relative quantitative RNA levels were calculated by the method of 2^−∆∆CT^.

### Western blot

Total proteins from lentivirus infected HO-8910 and OVCAR-3 cells were lysed by Lysis Buffer and the concentration was measured using BCA Protein Assay Kit (Cat. # 23225, HyClone-Pierce). After heated for 20 min at 100 °C, proteins were electrophoresed through a 10% SDS-PAGE. PVDF membranes were blocked by TBST with 5% skim milk, and then the membranes were incubated with primary antibodies and secondary antibody (detailed in Table S3) at 4 °C overnight. ECL plusTM Western blotting system kit from Amersham was used for color developing and target proteins detecting and bands were analyzed with ImageJ software.

### MTT assay

Cell proliferation of the infected HO-8910 and OVCAR-3 cells were detected through MTT assay. 2500 cells/well were seeded in 96-well plates and maintained for 24-120 h. MTT assay solution (20 μL, 5 mg/mL; Genview) was added into each well for 4 h reaction, and then 100 μL dimethyl sulfoxide (DMSO) were added to solve Formazan crystal. Cell proliferation was revealed by microplate reader (Tecan infinite) at 490/570 nm.

### FACS apoptosis assay

Infected HO-8910 and OVCAR-3 cells were harvested and centrifuged at 1300 rpm for 5 min, and the cell precipitation was washed with ice-cooled D-Hanks (pH=7.2-7.4). Then cells precipitation was suspended with 200 μL 1 × binding buffer and then cells were stained by 10 μL Annexin V-APC for 15 min in the dark. FACScan (Millipore) was used to assess the apoptosis rate.

### Colony formation assay

Infected OVCAR-3 and HO-8910 cells were cultured in a 6-well plate (500 cell/well) in the incubator for 14 days and the cell culture medium was changed every three days. The cell clones were photographed under fluorescence microscope at the end of the experiment After washing, cell clones were fixed with 4% paraformaldehyde for 60 min, and then stained with GIEMSA (DingGuo Biotechnology) for 15 min, digital camera. The clone number was counted (a clone with cells >50 cells were counted).

### Wound-healing assay

Briefly, lentivirus infected HO-8910 and OVCAR-3 cells (5 × 10^4^ cells/well) were seeded into a 96-well dish for 24 h culturing. When the cells confluence reached 90%, cell scratches across the cell layer were made with a 96 wounding replicator (VP scientific). The scratches were gently rinsed with PBS for 2-3 times. Photographs were taken at 8 h and 24 h using a fluorescence microscope (OLYMPUS).

### Transwell assay

The migration ability of lentivirus infected HO-8910 and OVCAR-3 cells was analyzed through Transwell assay. Cell suspension (8 × 10^4^ cells) was loaded into the serum-free medium upper chamber of the Transwell (Corning). The lower chamber was filled with 600 μL containing 30% FBS. After cultured for 24 h at 37 °C with 5% CO_2_, non-metastatic cells were washed. Metastatic cells were fixed by 4% formaldehyde and stained by 400 µL Giemsa. Transwell transfer rate was calculated.

### Celigo cell counting assay

Lentivirus infected HO-8910 cells and negative control cells were seeded in a 96-well plate with 2000 cells per well for culturing. Each group was set three wells. From the second day after seeding, cells were detected by Celigo (Nexcelom) once a day for 5 consecutive days. The number of cells with green fluorescence was calculated and cell proliferation rate was analyzed.

### Human apoptosis antibody array

Genes involved in human apoptosis signal pathway were valued by human apoptosis antibody array (Cat. #ab134001, Abcam) was applied following the manufacturer’s instructions. Each array membrane was blocked using Blocking Buffer, and prepared proteins (0.5 mg/mL) from lentivirus infected HO-8910 cells was incubated with blocked array membrane overnight at 4 °C. After washing, membrane was incubated with streptavidin-HRP was added to incubate at room temperature for 2 h. The signals were detected using enhanced chemiluminescence (ECL) and spots gray was analyzed by ImageJ software (National Institute of Health).

### GeneChip primeview human assay

Total RNA was extracted by TRIZOL Reagent (Life technologies) following the manufacturer’s instructions. RNA integrity was qualified by Agilent Bioanalyzer 2100 (Agilent technologies). Affymetrix human GeneChip 3′ IVT PLUS Reagent Kit was used to obtain biotin labeled cRNA in light of the manufacturer’s instruction. cRNA were hybridized with GeneChip Human array at 45 °C. Next, the array was washed and stained by Affymetrix Fluidics Station 450, and scanned by the Affymetrix GeneChip Scanner. The statistical significance assessment of raw data was accomplished through a Welch t-test with Benjamini–Hochberg FDR (<0.05 as significant). The significance test and functional analysis were conducted based on Ingenuity Pathway Analysis (IPA) (Qiagen). |Fold Change | > 2.0 and FDR < 0.05 was used for significant difference genes screening criteria and |Z score | > 2 is regarded as significant.

### Tumor-bearing mice model

0.2 mL cell suspension (4 × 10^6^ cells) of lentivirus infected logarithmic growth phase HO-8910 cells was injected subcutaneously into female BALB/c nude mice (4 week-old, grouped as shCtrl and shPSMC2), which were purchased from Beijing Vital River Laboratory Animal Technology Co., Ltd. Tumors volume and weight of mice was measured once per week. Before terminated the experiment, all mice were anesthetized by sodium pentobarbital (0.7%, 10 μL/g) intraperitoneal injection and placed under an IVIS Spectrum (Perkin Elmer) for imaging, and the fluorescence was observed and pictures were photographed. Then all mice were sacrificed and the tumors were used for Ki-67 immunostaining assay to detect the expression level in mice tumor tissues. All the animal experiments were approved by ethical committee of Shengjing Hospital of China Medical University.

### Statistical analysis

Each experiment was carried out at least 3 times under the same conditions. Data were showed as means and standard deviation (SD) for continuous data. Nonparametric data was expressed as percentage. Significant differences between groups were assessed with Student’s t-test or one-way ANOVA analysis using SPSS 21.0 Software or GraphPad Prism 6. Chi square test or Mann–Whitney *U* test for non-parametric data. *P* < 0.05 was considered statistically significant.

## Results

### PSMC2 is highly expressed in ovarian and indicates poor prognosis

**Fig. 1:**
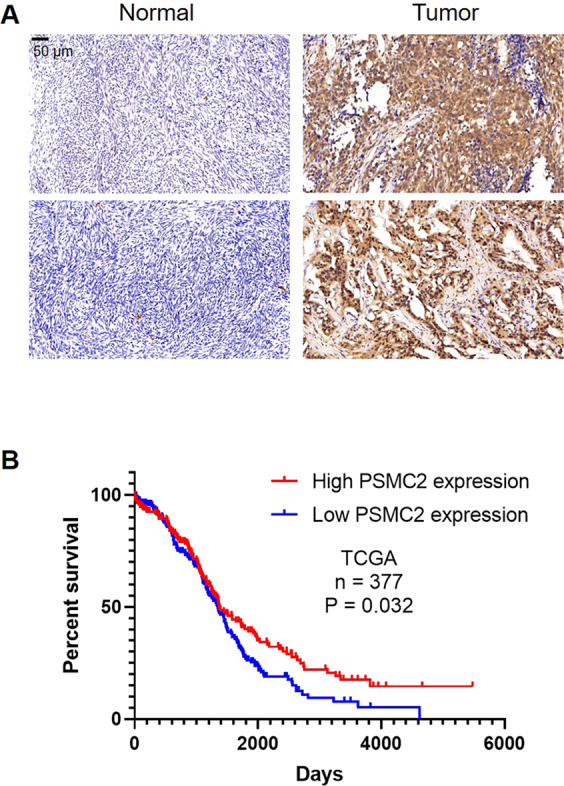
Upregulation of PSMC2 in ovarian cancer predicts poor prognosis. **A** IHC analysis was used to determine the expression levels of PSMC2 in ovarian cancer tissues and normal tissues. **B** PSMC2 expression and survival data were collected from TCGA database for conducting Kaplan–Meier survival analysis.

First of all, the expression of PSMC2 was evaluated in ovarian cancer tissues, followed by a comparison with that in normal tissues to initially investigate the possible involvement of PSMC2 in ovarian cancer. The outcomes of the IHC staining demonstrated the obvious upregulation of PSMC2 in ovarian cancer tissues (Fig. 1A and Table 1). The correlation analysis between PSMC2 and tumor characteristics showed significant association between PSMC2 expression and tumor grade (Table 2 and S4). Moreover, although no normal tissue samples are available in TCGA database, the survival analysis based on the features of 377 ovarian cancer patients built the linkage of PSMC2 high expression and poor prognosis of patients (Fig. 1B). Consequent upon findings mentioned, PSMC2 may have a critical regulatory function in the development and progression of ovarian cancer.

**Table 1:** Expression patterns of PSMC2 in ovarian cancer tissues and normal tissues revealed in immunohistochemistry analysis.

PSMC2 expression	Tumor tissue		Normal tissue	
	Cases	Percentage	Cases	Percentage
Low	56	37.8%	58	100%
High	92	62.2%	0	–

*P* < 0.001.

**Table 2:** Relationship between PSMC2 expression and tumor characteristics in patients with ovarian cancer.

Features	No. of patients	PSMC2 expression		*P* value
		Low	High	
All patients	148	56	92	
Age (years)				0.197
<52	74	32	42	
≥52	73	24	49	
Grade				0.019*
I	14	6	8	
II	17	10	7	
III	86	23	63	
T Infiltrate				0.715
T1	9	5	4	
T2	35	12	23	
T3	103	38	65	
Lymphatic metastasis (N)				0.897
N0	106	40	66	
N1	41	15	26	
Metastasize (M)				0.790
M0	114	42	72	
M1	33	13	20	
Stage				0.896
1	9	5	4	
2	35	12	23	
3	70	25	45	
4	33	13	20	
Tumor size				0.118
<12.5 cm	73	23	50	
≥12.5 cm	75	33	42	
Tumor recurrence				0.359
No	29	13	16	
Yes	118	42	76	

### PSMC2 promotes ovarian cancer cell proliferation and migration

**Fig. 2:**
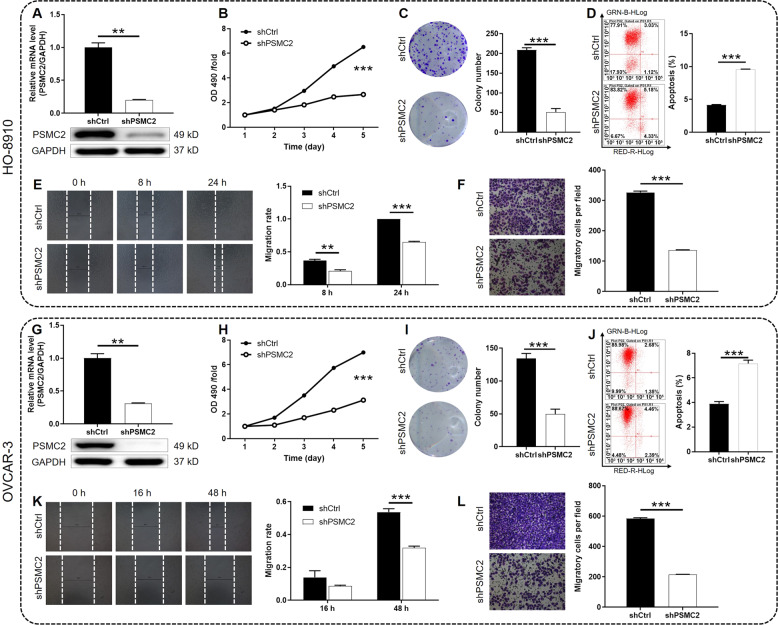
Knockdown of PSMC2 inhibits ovarian cancer development in vitro. **A**,, **G** qPCR and western blotting were used to confirm the construction of PSMC2 knockdown ovarian cancer cell lines. **B**, **H** The effects of PSMC2 on cell proliferation were evaluated by MTT assay. **C**, **I** The colony formation ability of shPSMC2 and shCtrl cells was assessed by colony formation assay. **D**, **J** The percentage of apoptotic cells was determined by flow cytometry assay. **E**, **K** Wound-healing assay was performed to show the cell migration ability of ovarian cancer cells transfected with shPSMC2 or shCtrl. **F**, **L** Transwell assay was carried out to show the cell migration ability of ovarian cancer cells transfected with shPSMC2 or shCtrl. Data were presented as mean ± standard deviation based on at least 3 independent experiments. **P < 0.01, ***P < 0.001.

Next, we carried out a loss-of-function study through downregulating the endogenous expression of PSMC2 in ovarian cancer cell lines HO-8910 and OVCAR-3 based on lentivirus criteria. Lentivirus expressing shPSMC2 was applied for constructing PSMC2 knockdown cell lines and that contains shCtrl was used to prepare the negative control cell lines. The validity of PSMC2 silencing was verified by qPCR and western blotting, respectively, which indicated that PSMC2 was significantly knocked down in shPSMC2 cells in comparison with shCtrl cells (Fig. 2A and G, *P* < 0.01). MTT assay was conducted to assess the influences of PSMC2 on cell proliferation, indicating the significantly suppressed cell growth of shPSMC2 cells (Fig. 2B and H, *P* < 0.001). Consistently, the weakened colony-forming capacity of shPSMC2 cells was also displayed through colony formation assay (Fig. 2C and I). Subsequently, flow cytometry was performed to assess cell apoptosis and cell cycle distribution as an explanation of the regulatory effects on cell proliferation. As expected, cell apoptosis rate of shPSMC2 cells was apparently higher than that of shCtrl cells (Fig. 2D and J, *P* < 0.001). Meanwhile, knockdown of PSMC2 induced the arrest of cell cycle in G2 phase (Fig. S1, *P* < 0.05). Moreover, PSMC2’s effects on cell migration were estimated by wound-healing and Transwell assays, respectively. The wound healing rate (Fig. 2E and K, *P* < 0.001 at 24 h for HO-8910 or at 48 h for OVCAR-3 cells) and migratory cell numbers (Fig. 2F and L, *P* < 0.001) of shPSMC2 were significantly higher than shCtrl cells. Collectively, knockdown of PSMC2 caused a loss in cell growth and cell migration ability of ovarian cancer cells.

### PSMC2 regulates ovarian cancer through regulating apoptosis-related proteins and Akt pathway

**Fig. 3:**
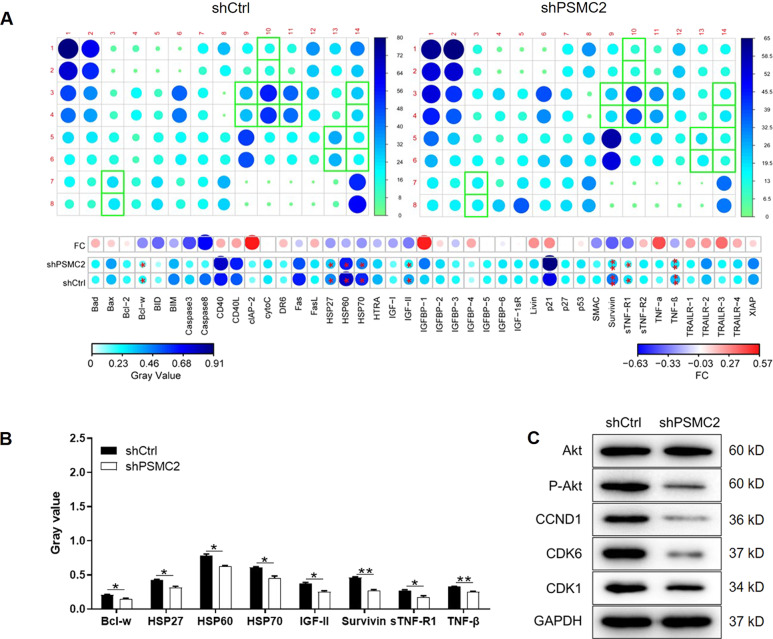
PSMC2 regulates ovarian cancer cell apoptosis through regulating apoptosis-related proteins and Akt pathway. **A** An apoptosis antibody array was used to detect the differential expression of 43 apoptosis-related proteins in shPSMC2 and shCtrl cells. **B** 8 of the 43 apoptosis-related proteins were significantly downregulated in shPSMC2 cells. **C** The expression of Akt, p-Akt, CCND1, CDK6 and CDK1 in shPSMC2 and shCtrl cells was detected by western blotting. Data were presented as mean ± standard deviation based on at least 3 independent experiments. *P < 0.05, **P < 0.01.

For exploring the mechanism by which PSMC2 regulated cell growth and cell apoptosis, an apoptosis antibody array was used to evaluate the difference of expression levels of apoptosis-related proteins in shPSMC2 and shCtrl cells. As shown in Fig. 3A and B, 43 proteins were tested and 8 of them, including Bcl-w, HSP27, HSP90, IGF-II, Survivin, sTNF-R1 and TNF-β, exhibited significantly downregulated levels (Fig. 3C). Moreover, the enrichment of these proteins in Akt signaling pathway determined by KEGG make us believe that Akt pathway may be involved. Therefore, the levels of Akt and p-Akt, as well as some downstream factors such as CCND1, CDK6 and CDK1 were also examined in shPSMC2 and shCtrl cells. The results of western blotting showed the lowered activity of Akt as well as downregulated expression of p-Akt, and the lower expression of CCND1, CDK6 and CDK1 in shPSMC2 cells (Fig. 3D).

### PSMC2 regulates tumor growth of ovarian cancer in vivo

**Fig. 4:**
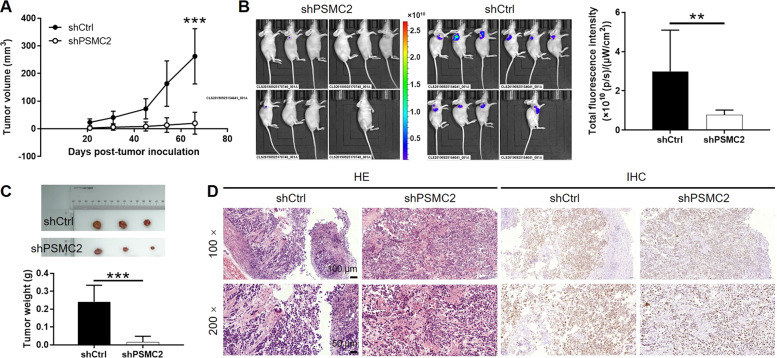
Knockdown of PSMC2 inhibits tumor growth of ovarian cancer in vivo. HO-8910 cells transfected with shPSMC2 or shCtrl were used for constructing mouse xenograft models. The size of xenografts was measured and the volume of them was calculated correspondingly throughout the animal experiments (**A**). Before sacrificing the mice, they were subjected to in vivo imaging for observing the growth of xenograft in situ (**B**). After sacrificing the mice, the xenografts were removed for taking photos and weighing (**C**). Finally, the Histological characteristics and Ki67 levels in sections of xenografts were determined by HE and IHC staining, respectively (**D**). Data were presented as mean ± standard deviation (10 mice in each group). **P < 0.01, ***P < 0.001.

For further exploring the role of PSMC2 in the development of ovarian cancer, we injected HO-8910 cells transfected with shPSMC2 or shCtrl subcutaneously into nude mice for developing xenografts. The continuous measurement of tumor size and volume showed that xenografts developed from shPSMC2 cells grew much slower than that derived from shCtrl cells (Fig. 4A). In vivo imaging facilitated by GFP label on lentiviral vector also demonstrate much higher tumor burden in shCtrl group than shPSMC2 group (Fig. 4B). After sacrificing the animal models, xenografts were removed and collected for taking photos, weighing, H&E staining and IHC analysis. The outcomes of these tests exhibited that xenografts formed by shPSMC2 cells was smaller and lighter, as well as possessed lower Ki67 level in comparison with shCtrl group (Figs. 4C, D and S2). All these demonstrated that knockdown of PSMC2 could suppress tumor growth in vivo.

### PSMC2 promotes ovarian cancer through regulating CCND1

**Fig. 5:**
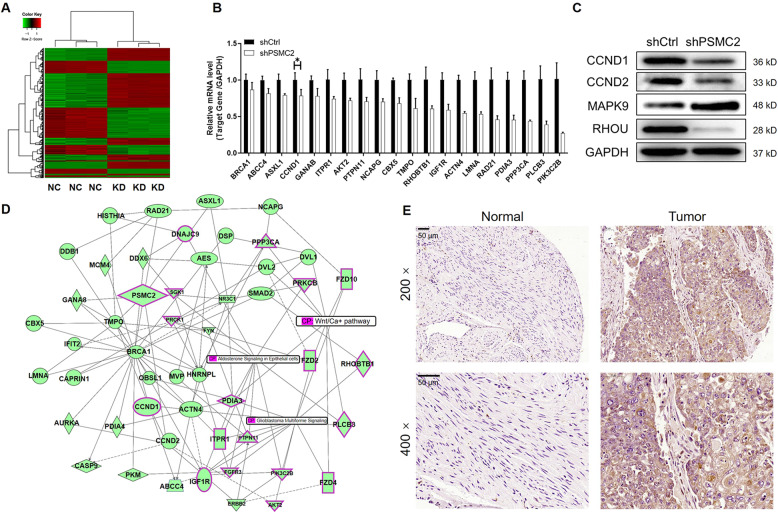
PSMC2 regulates ovarian cancer development through CCND1. **A** Microarray analysis was performed to identify the upregulated and downregulated genes in shPSMC2 cells. **B**, **C** Several downregulated DEGs were selected for qPCR (B) and western blotting (**C**) verification. **D** A PSMC2-centered molecular interaction network established based on IPA displayed the potential interaction between PSMC2 and CCND1. **E** The expression of CCND1 in ovarian cancer tissues and normal tissues was detected by IHC. Data were presented as mean ± standard deviation based on at least 3 independent experiments. *P < 0.05.

As mechanistic study, a microarray analysis was made on HO-8910 cells transfected with shPSMC2 or shCtrl, identifying 846 upregulated differentially expressed genes (DEGs) and 736 downregulated DEGs (Figs. 5A and S3A). The enrichment analysis based on IPA showed the most significant enrichment of DEGs in p53 signaling pathway and the distinct enrichment in functions such as cancer and cell cycle (Figs. S3B and S3C). More obviously, the downregulation of mRNA and protein levels of CCND1 in shPSMC2 cells was demonstrated in microarray analysis, qPCR and western blotting (Figs. 5B and C), which was in keeping with our previous results in Fig. 3C. Moreover, PSMC2 and CCND1 could also be linked during the construction of molecular interaction network using the results of microarray analysis (Fig. 5D). Furthermore, we also tested the expression of CCND1 in ovarian cancer tissues and corresponding normal tissues, indicating an upregulation of CCND1 in ovarian cancer (Fig. 5E). Bearing all these in mind, we hypothesized that CCND1 may serve as a potential downstream target of PSMC2 in the regulation of ovarian cancer.

### PSMC2 promotes ovarian cancer development through CCND1

**Fig. 6:**
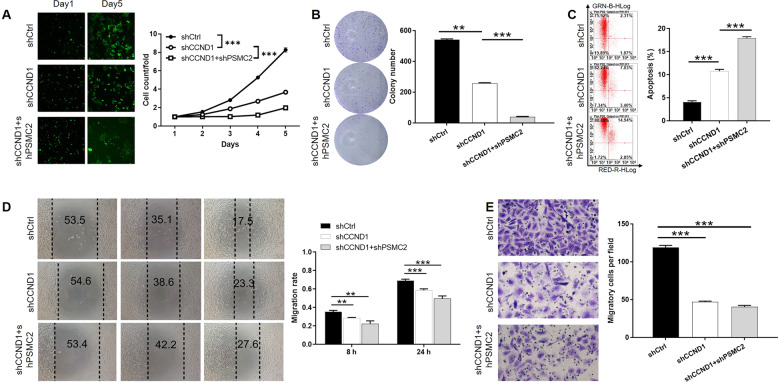
Knockdown of PSMC2 and CCND1 synergistically inhibits ovarian cancer development in vitro. **A** MTT assay was applied to evaluate the impacts of CCND1 knockdown alone or PSMC2 and CCND1 knockdown together on HO-8910 cell proliferation. **B** The effects of CCND1 knockdown alone or PSMC2 and CCND1 knockdown together on colony formation ability of HO-8910 cells were assessed. **C** The regulation of cell apoptosis by CCND1 knockdown alone or PSMC2 and CCND1 knockdown together was detected by flow cytometry. Wound-healing (**D**) and transwell (**E**) assays were utilized for determining the impacts of CCND1 knockdown alone or PSMC2 and CCND1 knockdown together on cell migration. Data were presented as mean ± standard deviation based on at least 3 independent experiments. **P < 0.01, ***P < 0.001.

Although the role of CCND1 in ovarian cancer has been well described, its synergistic effect with PSMC2 has not been investigated. Therefore, HO-8910 cells were transfected with shCtrl or shCCND1 (shCCND1-1, Fig. S4) alone or with shPSMC2 and shCCND1 together and the knockdown of PSMC2 and CCND1 was verified by qPCR (Fig. S5A). Interestingly, it was demonstrated by western blotting that protein level of PSMC2 was downregulated in shCCND1 cells and vice versa, indicating the mutual regulation between them (Fig. S5B). More importantly, the detection of malignant phenotypes of HO-8910 cells suggested that the inhibition of cell proliferation, colony formation and the promotion of cell apoptosis could be significantly amplified by silencing PSMC2 and CCND1 together (Fig. 6A–C). Not surprisingly, the transfection of shPSMC2 + shCCND1 exhibited the strongest ability to inhibit cell motility, which was visualized by both wound-healing and Transwell assays (Fig. 6D–E). Moreover, we further overexpressed CCND1 in shPSMC2 cells and detected the cell apoptosis. The results showed that CCND1 overexpression could reverse the promotion of cell apoptosis by PSMC2 knockdown (Fig. S6). Altogether, we believe that PSMC2 may regulate the development of ovarian cancer in combination of CCND1.

## Discussion

In the current study, we identified PSMC2 as a critical regulator of the ovarian cancer development and progression. Ovarian cancer tissues possess relatively higher expression of PSMC2 than normal tissues. High PSMC2 expression also predicts more advanced tumor grade of ovarian cancer. Data collected from TCGA database also potentiate the relationship between PSMC2 level and poor prognosis of patients with ovarian cancer. Loss-of-function assays performed on ovarian cancer cells with or without PSMC2 knockdown illustrated that PSMC2 may promote the progress of ovarian cancer by means of promoting cell proliferation, colony formation, cell migration and alleviating cell apoptosis. Moreover, xenografts formed by ovarian cancer cells with PSMC2 grow slower and present lower Ki67 index, which agrees with the in vitro results. As one of the essential components of 19 S regulatory granules, PSMC2 has the functions of ATP binding, nucleotide binding, nucleoside triphosphatase and hydrolase and is mainly involved in regulating the selective degradation of intracellular proteins. Despite, little attention has been paid to the tumor regulatory functions of PSMC2. Qin et al. utilized a loss-of-function strategy to reveal the potential role of PSMC2 as a tumor promotor in pancreatic cancer, which was highly expressed in pancreatic cancer tissues and capable of inhibiting cell proliferation and enhancing cell apoptosis [18, 19]. He et al. reported that PSMC2 may also play an important role in colorectal cancer because tumors with higher malignancy, as well as poorer prognosis, were frequently accompanied with higher PSMC2 expression. They also proved the in vitro inhibition of colorectal cancer by PSMC2 knockdown [20]. Actually, similar functions of PSMC2 in osteosarcoma were also discovered by Song et al. [19]. All these outcomes indicated that PSMC2 may be a wide-range tumor promotor in human cancers.

In the mechanistic study on PSMC2-induced cell apoptosis, Bcl-w, which has been well-documented to be a mediator in the regulation of ovarian cancer [21], was found to be downregulated upon PSMC2 knockdown. Heat shock proteins have been well-known to be protectors against different types of cell death, especially apoptosis and necrosis, which also is considered to have important role in development of ovarian cancer and as therapeutic opportunities [22–24]. Herein, we also found the decrease of HSP27, HSP60 and HSP70 in shPSMC2 cells, which may be the reason of apoptosis enhancement. Moreover, well-known anti-apoptosis proteins in ovarian cancer including IGF-II [25] and Survivin [26] were also found to be declined in shPSMC2 cells.

CCND1, also known as cyclin D1, was identified as a potential downstream of PSMC2, which regulate the development of ovarian cancer in together with PSMC2. The results of microarray, qPCR and western blotting all demonstrated that CCND1 could be downregulated by PSMC2 knockdown. The combination of shPSMC2 and shCCND1 induced stronger impacts on cell proliferation, colony formation, cell apoptosis and cell migration than shPSMC2 or shCCND1 alone. The cell cycle process is driven by a complex formed by a variety of different cyclins and cyclin-dependent kinases (CDKs). Through the regulation of cyclins/CDKs, the cells can precisely regulate every phase of the cell cycle to ensure the normal growth and development. In the G1 phase of the cell cycle, cyclin D is highly expressed. The cyclin D family includes D1, D2 and D3, which can sense signal stimulation inside and outside the cells, bind and activate the cyclin-dependent kinase CDK4/6 to initiate the cell cycle [27]. Moreover, in terms of quantity and function, CCND1 occupies a dominant position among cyclin D family. It is regarded as a mitogenic cell sensor, and its abnormal expression can lead to cell cycle disorders and cell dysfunction [28]. Therefore, CCND1 has attracted extensive and in-depth attention and research in the field of tumor research [29, 30]. In fact, numerous studies have shown that the amplification of CCND1 is a common phenomenon in human cancers [31–33]. Specifically, Dai et al. presented the role of CCND1 in the promotion of ovarian cancer cell proliferation, which could be alleviated by the treatment of cisplatin [34]. Study of Zhong et al. showed that depletion of cyclin D1 increased the sensitivity of ovarian cancer cells to olaparib through disturbing RAD51 accumulation and inducing cell cycle G0/G1 arrest [34, 35]. Our study further indicated that the combined inhibition targeting PSMC2 and CCND1 may be an effective treatment strategy for ovarian cancer. Moreover, we also found that CDK6, as well as the Akt signaling, was downregulated together with CCND1 in shPSMC2 cells.

In conclusion, a novel tumor promotor PSMC2 was identified in ovarian cancer, which is upregulated in ovarian cancer, accelerating cell proliferation and colony formation, promoting cell migration, inhibiting cell apoptosis and predicting poor prognosis. In addition, PSMC2 may regulate ovarian cancer through CCND1, knockdown of both in combination could induce intense inhibition effects on the development and progression of ovarian cancer.

## Supplementary information

Table S3

Figure S1

Figure S2

Figure S3

Figure S4

Figure S5

Figure S6

Table S1

Table S2

Table S4

Supplementary figure legends

## Data Availability

All data generated or analyzed during this study are included in this published article and its supplementary information files.
