# Supplementary figures and images for: PSMC2/CCND1 axis promotes development of ovarian cancer through regulating cell growth, apoptosis and migration
Source: Cell Death Dis. 2021 Jul 22;12(8):730. doi: 10.1038/s41419-021-03981-5 (PMC8298468; doi:10.1038/s41419-021-03981-5)

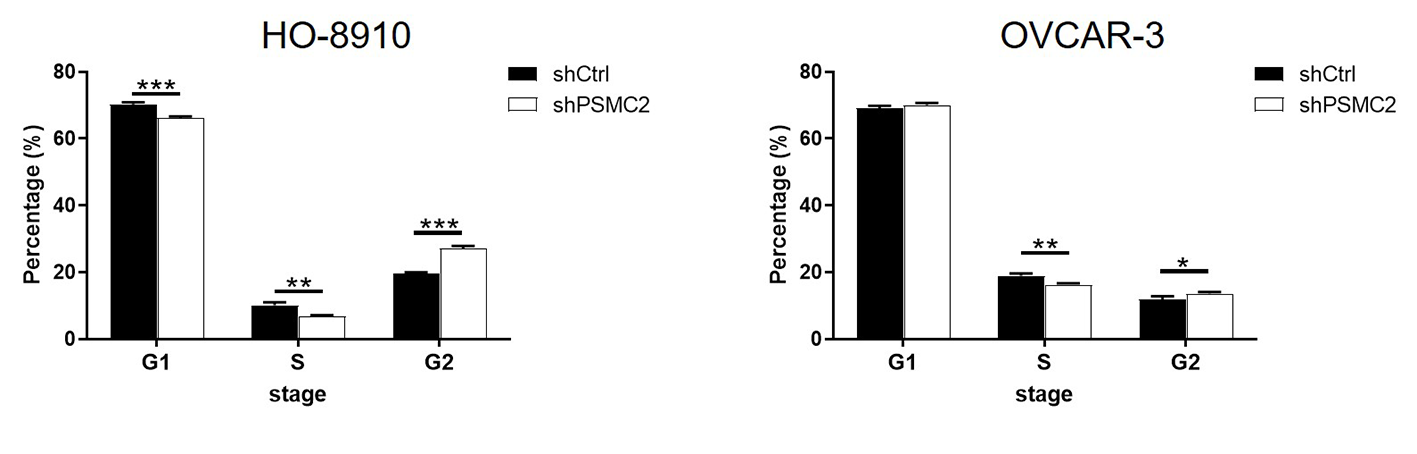

Supplement: Supplementary file 2 — Figure S1 [file 41419_2021_3981_MOESM2_ESM.tif]

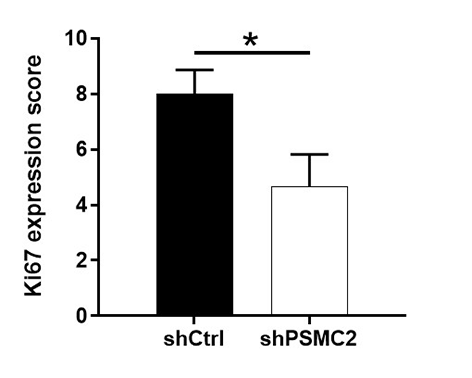

Supplement: Supplementary file 3 — Figure S2 [file 41419_2021_3981_MOESM3_ESM.tif]

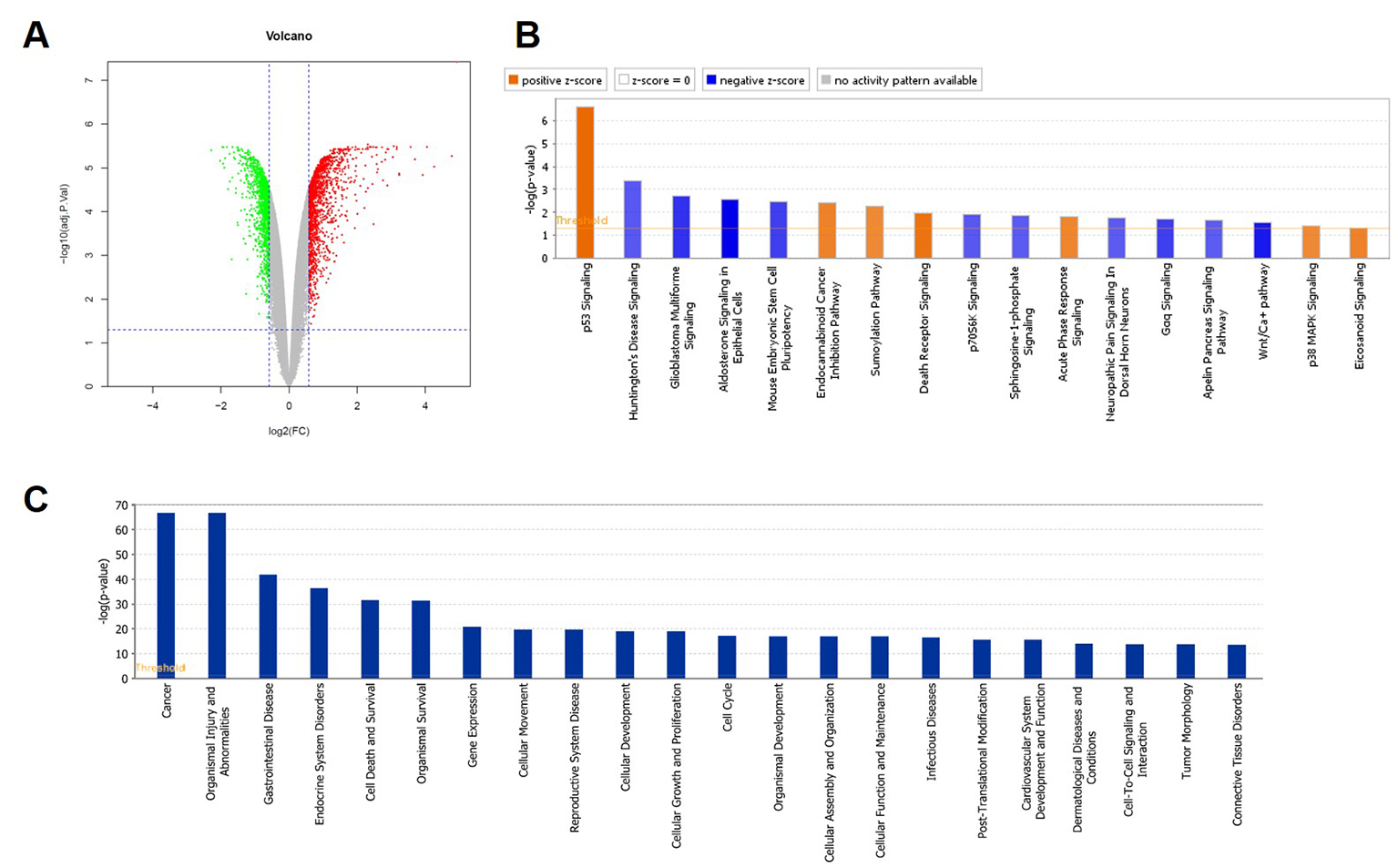

Supplement: Supplementary file 4 — Figure S3 [file 41419_2021_3981_MOESM4_ESM.tif]

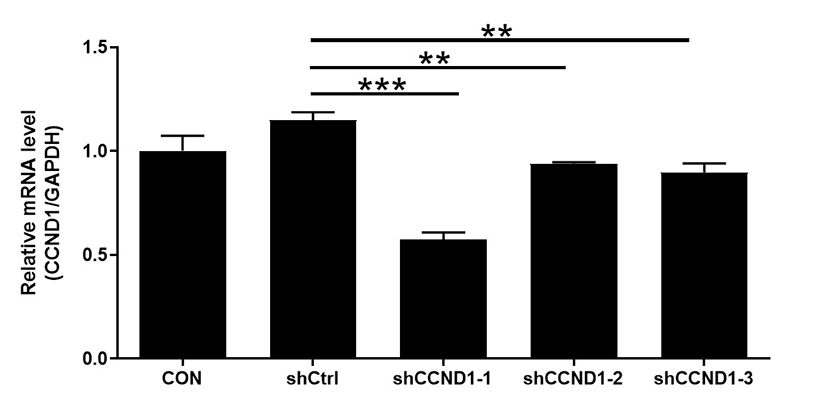

Supplement: Supplementary file 5 — Figure S4 [file 41419_2021_3981_MOESM5_ESM.tif]

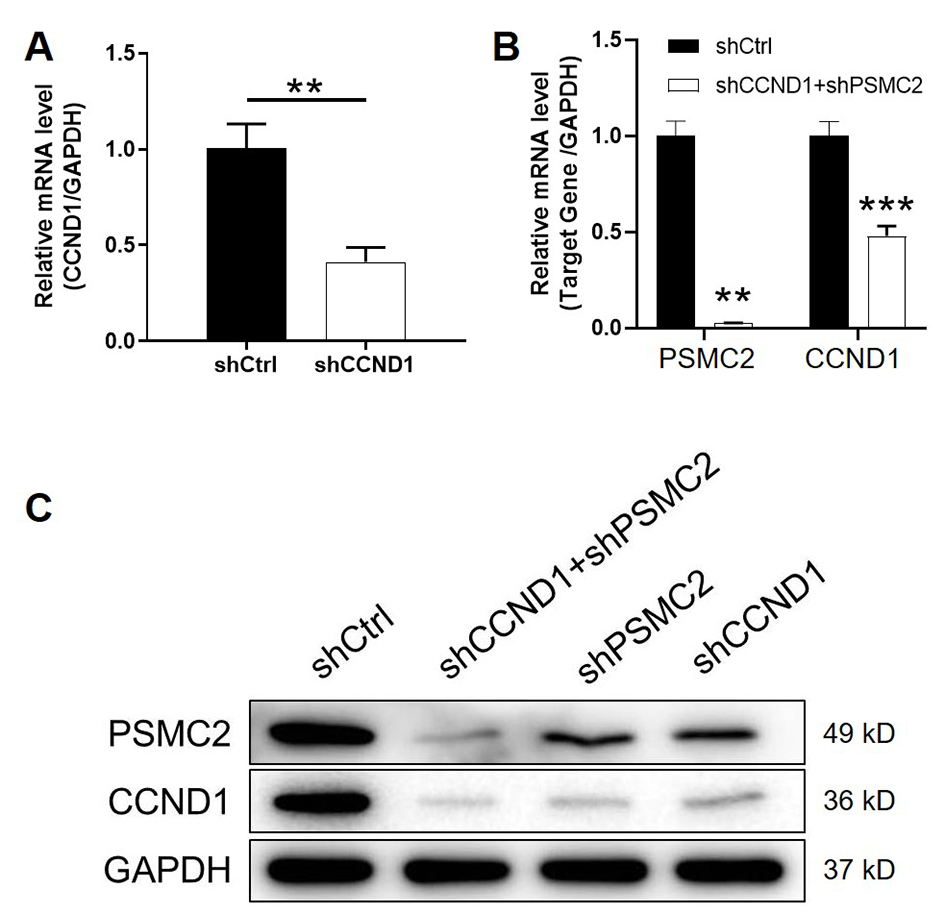

Supplement: Supplementary file 6 — Figure S5 [file 41419_2021_3981_MOESM6_ESM.tif]

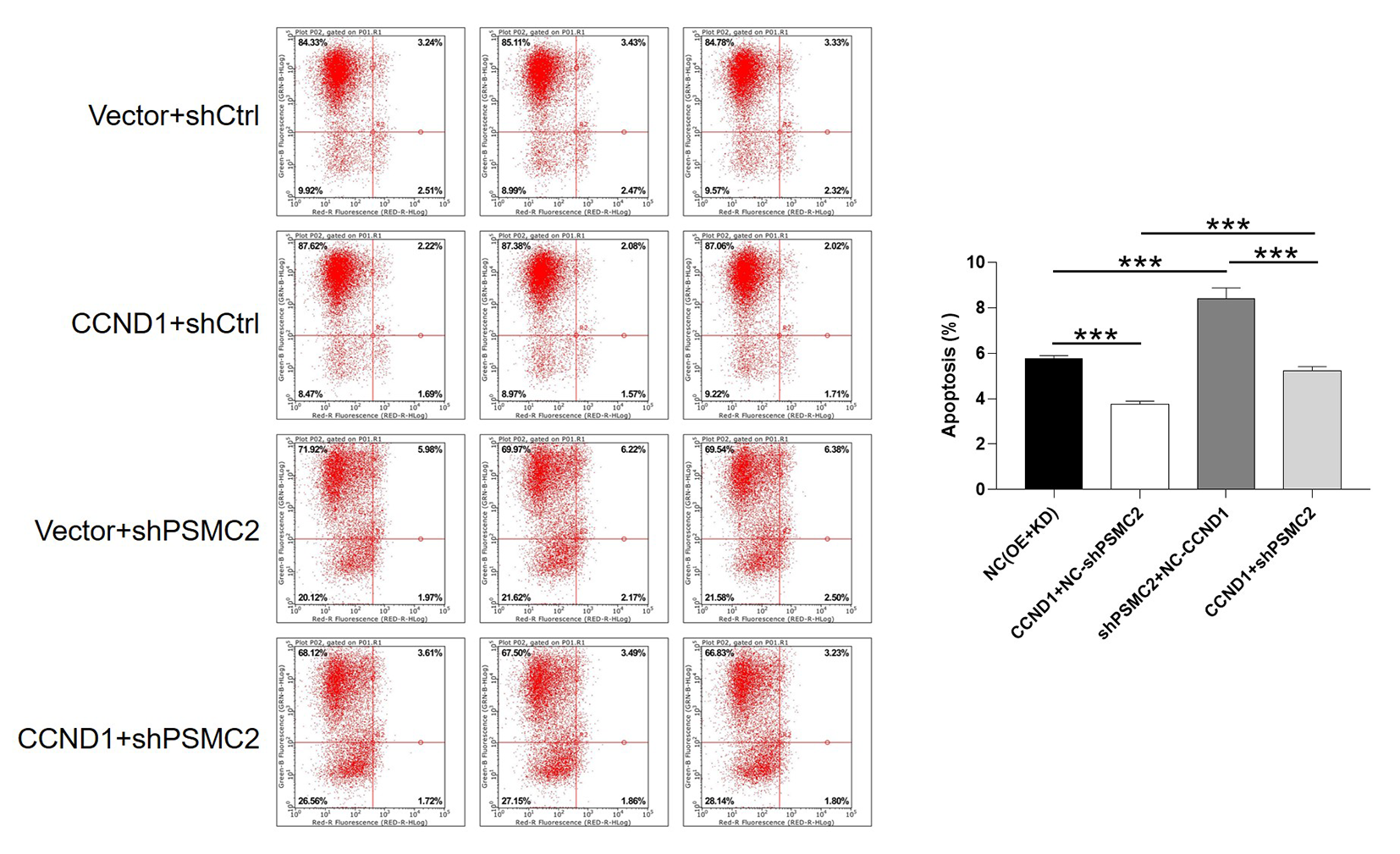

Supplement: Supplementary file 7 — Figure S6 [file 41419_2021_3981_MOESM7_ESM.tif]
