# Supplementary material for: PSMC2/CCND1 axis promotes development of ovarian cancer through regulating cell growth, apoptosis and migration
Source: Cell Death Dis. 2021 Jul 22;12(8):730. doi: 10.1038/s41419-021-03981-5 (PMC8298468; doi:10.1038/s41419-021-03981-5)
Supplement: Supplementary file 8 — Table S1 [file 41419_2021_3981_MOESM8_ESM.docx]

Table S1 The target sequences and shRNA sequences

| Gene | No. | Target sequence (5'-3') | shRNA sequences (5'-3') |
| --- | --- | --- | --- |
| PSMC2 | Pbr-00145-a | GCCAGGGAGATTGGATAGAAA | CcggGCCAGGGAGATTGGATAGAAATTCAAGAGATTTCTATCCAATCTCCCTGGCTTTTTg |
| PSMC2 | Pbr-00145-b | GCCAGGGAGATTGGATAGAAA | aattcaaaaaGCCAGGGAGATTGGATAGAAATTCAAGAGATTTCTATCCAATCTCCCTGGC |
| PSMC2 | Pbr-23881-a | CAACGTAAAGCAGTTTGCCAA | CcggCAACGTAAAGCAGTTTGCCAActcgagTTGGCAAACTGCTTTACGTTGTTTTTg |
| PSMC2 | Pbr-23881-b | CAACGTAAAGCAGTTTGCCAA | aattcaaaaaCAACGTAAAGCAGTTTGCCAActcgagTTGGCAAACTGCTTTACGTTG |
| PSMC2 | Pbr-23882-a | AAGCAAGTTGAAGATGACATT | CcggAAGCAAGTTGAAGATGACATTctcgagAATGTCATCTTCAACTTGCTTTTTTTg |
| PSMC2 | Pbr-23882-b | AAGCAAGTTGAAGATGACATT | aattcaaaaaAAGCAAGTTGAAGATGACATTctcgagAATGTCATCTTCAACTTGCTT |
| CCND1 | Pbr10190-a | GGTGAACAAGCTCAAGTGGAA | CCGGGGTGAACAAGCTCAAGTGGAACTCGAGTTCCACTTGAGCTTGTTCACCTTTTTG |
| CCND1 | Pbr10190-b | GGTGAACAAGCTCAAGTGGAA | AATTCAAAAAGGTGAACAAGCTCAAGTGGAACTCGAGTTCCACTTGAGCTTGTTCACC |
| CCND1 | Pbr00221-a | CCTCGGTGTCCTACTTCAAAT | CCGGCCTCGGTGTCCTACTTCAAATCTCGAGATTTGAAGTAGGACACCGAGGTTTTTG |
| CCND1 | Pbr00221-b | CCTCGGTGTCCTACTTCAAAT | AATTCAAAAACCTCGGTGTCCTACTTCAAATCTCGAGATTTGAAGTAGGACACCGAGG |
| CCND1 | Pbr00222-a | CCTCTGTGCCACAGATGTGAA | CCGGCCTCTGTGCCACAGATGTGAACTCGAGTTCACATCTGTGGCACAGAGGTTTTTG |
| CCND1 | Pbr00222-b | CCTCTGTGCCACAGATGTGAA | AATTCAAAAACCTCTGTGCCACAGATGTGAACTCGAGTTCACATCTGTGGCACAGAGG |
|  |  |  |  |
