# Supplementary material for: PSMC2/CCND1 axis promotes development of ovarian cancer through regulating cell growth, apoptosis and migration
Source: Cell Death Dis. 2021 Jul 22;12(8):730. doi: 10.1038/s41419-021-03981-5 (PMC8298468; doi:10.1038/s41419-021-03981-5)
Supplement: Supplementary file 9 — Table S2 [file 41419_2021_3981_MOESM9_ESM.docx]

Table S2 Primers used in qPCR

| Gene | Forward primer sequence (5’-3’) | Reverse primer sequence (5’-3’) |
| --- | --- | --- |
| GAPDH | TGACTTCAACAGCGACACCCA | CACCCTGTTGCTGTAGCCAAA |
| CCND1 | AGCTGTGCATCTACACCGAC | GAAATCGTGCGGGGTCATTG |
| PSMC2 | CAGCACTCTGGGATTTGGCT | TTTCTATCCACGCCCACTCTC |
| IGF1R | AAGTGACGGGGACTAAAGGG | GGTGCCAGGTTATGATGATGC |
| ABCC4 | GAAAGTGCCAAAGTAATCCAGC | GTGTTCAAAGCCACAGAATCCA |
| ITPR1 | GTTGTTCAGCACTTCGTTCACT | CTCGGCCATAACCATGTCTT |
| ACTN4 | CCCATGACCAGTTCAAGTCC | CGACAGCTTGATGTGGTTGCT |
| LMNA | ATCGCTTGGCGGTCTACAT | TGACCACCTCTTCAGACTCGGT |
| AKT2 | CCTCATGCTGGACAAAGATGG | GGGGTCCCACAGAAGGTTTT |
| NCAPG | GAAGAAGGTGACTGGGGAACT | CTGCTCTAACAATGGGTGGC |
| ASXL1 | GGCAGAAGGACTAAAGGAAATG | TGAGCGTGAAAAGGCTGATT |
| PDIA3 | GCTGGGCACAAACTCAACTT | GAACTCCTCCTGCATGACAAA |
| BRCA1 | TGGCAACATACCATCTTCAACC | TGTCAATTCTGGCTTCTCCCT |
| PIK3C2B | CGCTATGGCAACCGAAAGA | GGCAAAATGCAGCAACCTC |
| CBX5 | CTTCAGAGGATGAGGAGGAGTATG | GCTCAGGGCAATCCAAGTTTT |
| PLCB3 | TGAGCAATGGGGAGGAGGTA | AGCAGGTCCAAGAACATAGGG |
| CCND1 | AGCTGTGCATCTACACCGAC | GAAATCGTGCGGGGTCATTG |
| PPP3CA | TGCCTGTATGGATGCCTTTG | GGTGGTTCTTTGAATCGGTCTA |
| PTPN11 | CGGCAAGTCTAAAGTGACCC | AATCAAACCGTTCTCCTCCAC |
| TMPO | CCCCTATGAAGCATCTACACCA | GCCAAGGGAACATACTTAGGAAC |
| RAD21 | CAGTGCTTTTGAGGATGACGA | CTCATTCAGATTGCTGGTGCT |
| RHOBTB1 | GCACATCGAATTTACCTCGCTAC | TTCTCACAGGCTCCTTCACTCC |
| GANAB | GGGTGCTTACCAGCCATTCT | CCCAAGGCATCTCGGATTA |
