# Supplementary material for: PSMC2/CCND1 axis promotes development of ovarian cancer through regulating cell growth, apoptosis and migration
Source: Cell Death Dis. 2021 Jul 22;12(8):730. doi: 10.1038/s41419-021-03981-5 (PMC8298468; doi:10.1038/s41419-021-03981-5)
Supplement: Supplementary file 10 — Table S4 [file 41419_2021_3981_MOESM10_ESM.docx]

Table S4 Relationship between PSMC2 expression and tumor characteristics in patients with ovarian cancer analyzed by Pearson correlation analysis

| Tumor characteristics | index |  |
| --- | --- | --- |
| Grade | Pearson correlation | 0.217 |
|  | Significance (two tailed) | 0.019* |
|  | n | 117 |
