# Supplementary material for: PSMC2/CCND1 axis promotes development of ovarian cancer through regulating cell growth, apoptosis and migration
Source: Cell Death Dis. 2021 Jul 22;12(8):730. doi: 10.1038/s41419-021-03981-5 (PMC8298468; doi:10.1038/s41419-021-03981-5)
Supplement: Supplementary file 11 — Supplementary figure legends [file 41419_2021_3981_MOESM11_ESM.docx]

**Figure S1.** The effects of PSMC2 knockdown on cell cycle distribution were evaluated by flow cytometry. Data were presented as mean ± standard deviation based on at least 3 independent experiments. * P < 0.05, **P < 0.01, ***P < 0.001

**Figure S2.** The Ki67 expression scores of xenografts collected from shPSMC2 and shCtrl groups showed that Ki67 expression is higher in shCtrl group.

**Figure S3.** (A) A volcano plot of gene expression profiles in HO-8910 cells with or without PSMC2 knockdown. The green dots represent the downregulated DEGs. The red dots represent the upregulated DEGs. (B) The enrichment of the DEGs in canonical signaling pathways was analyzed by IPA. (C) The enrichment of the DEGs in IPA disease and function was analyzed by IPA.

**Figure S4.** The knockdown efficiencies of 3 shRNAs for silencing CCND1 were verified by qPCR. Data were presented as mean ± standard deviation based on at least 3 independent experiments. **P < 0.01, ***P < 0.001

**Figure S5.** The expression of PSMC2 and CCND1 in shCtrl, shCCND1, shPSMC2+shCCND1 groups of HO-8910 cells were evaluated through qPCR and western blotting. Data were presented as mean ± standard deviation based on at least 3 independent experiments. **P < 0.01, ***P < 0.001

**Figure S6.** The effects of mere CCND1 overexpression, mere PSMC2 knockdown and simultaneous CCND1 overexpression + PSMC2 knockdown on HO-8910 cell apoptosis were evaluated by flow cytometry. Data were presented as mean ± standard deviation based on at least 3 independent experiments. ***P < 0.001
